# Assessment of Bias in Patient Safety Reporting Systems Categorized by Physician Gender, Race and Ethnicity, and Faculty Rank: A Qualitative Study

**DOI:** 10.1001/jamanetworkopen.2022.13234

**Published:** 2022-05-20

**Authors:** Élan Burton, Brenda Flores, Barbara Jerome, Michael Baiocchi, Yan Min, Yvonne A. Maldonado, Magali Fassiotto

**Affiliations:** 1 Department of Cardiothoracic Surgery, Stanford University School of Medicine, Palo Alto, California; 2 Office of Faculty Development and Diversity, Stanford University School of Medicine, Palo Alto, California; 3 Department of Epidemiology and Population Health, Stanford University School of Medicine, Palo Alto, California

## Abstract

**Importance:**

Patient safety reporting systems (PSRSs) are designed to decrease the risk of harm to patients due to medical errors. Owing to the voluntary nature of PSRSs, implicit bias of the reporter may affect the management of safety events reported. Stanford Alert For Events (SAFE) is the PSRS used at Stanford Health Care.

**Objective:**

To examine whether variation exists in the content of SAFE reports based on demographic characteristics of physicians who are the subject of the event report.

**Design, Setting, and Participants:**

This retrospective qualitative analysis from a single academic medical center evaluated SAFE reports from March 2011 to February 2020. Event reports were coded by theme and categorized by severity (scale of 1 to 3, with 1 being the lowest and 3 the highest). The reports were then analyzed from October 2020 to February 2022 and categorized by physician gender, race and ethnicity, and faculty rank. A total of 501 patient safety events were collected from the adult hospital during the study period, and 100 were excluded owing to incompleteness of information.

**Main Outcomes and Measures:**

This qualitative study had no planned outcome.

**Results:**

A qualitative analysis was performed on 401 reports representing 187 physicians (138 [73.8%] male and 49 [26.2%] female). In terms of race and ethnicity, 4 physicians (2.1%) were African American, 49 (26.2%) were Asian; 7 (3.7%), Hispanic or Latinx; 108 (57.7%), White; and 19 (10.2%), declined to state. Female physicians had disproportionate representation among reports referencing communication and conversational issues and the lowest severity level. Male physicians had disproportionate representation for ignoring or omitting procedures, process issues, and physical intimidation. African American physicians had disproportionate representation for lack of communication and process issues. Asian physicians had disproportionate representation for lack of communication, process issues, conversational conduct, and the lowest severity level. Latinx physicians had disproportionate representation for conversational conduct. White physicians had disproportionate representation for ignoring or omitting procedures, verbal abuse, physical intimidation, and the highest severity level.

**Conclusions and Relevance:**

In this qualitative study, female physicians and physicians who were members of racial and ethnic minority groups were more likely to be reported for low-severity communication issues compared with their male and White counterparts, respectively. These findings suggest that there may be a lower threshold for reporting events when the subject of the report is female and/or a member of a racial or ethnic minority group. Restructuring the reporting and management of patient safety events may be needed to facilitate conflict resolution in a manner that reduces implicit bias and fosters team cohesion.

## Key Points

**Question:**

Does variation exist in the content of patient safety reports based on the demographic characteristics of the physicians who are the subject of the event report?

**Findings:**

In this qualitative study of 401 patient safety reports, physicians who were female or members of racial and ethnic minority groups were more likely to be reported for low-severity communication issues compared with their male and White counterparts, respectively.

**Meaning:**

These findings suggest that bias may be present in patient safety reporting systems, which could have repercussions for the career trajectories and/or employee working relationships of female physicians and physicians who are members of racial and ethnic minority groups.

## Teaser

This qualitative study assesses whether bias exists in patient safety reporting systems based on the gender, race and ethnicity, or faculty rank of the physician who is the subject of the event report.

## Introduction

The modern patient safety movement was initiated in 1999 with the release of the Institutes of Medicine report *To Err Is Human*.^1^ Since the release of this report, prevention of medical errors has been pushed to the forefront of health care, with patient safety reporting systems (PSRSs) now commonplace in medical institutions. The purpose of a PSRS is “to identify and mitigate risks to patients who are harmed by medical care.”^2^^(p1)^ Furthermore, PSRSs have evolved to identify disruptive behavior exhibited by care teams that could compromise patient safety. Although PSRSs aid in improving patient safety, they rely on voluntary reporting and may have varying efficacy rates. Hutchinson et al^3^ found no association between reporting rates and standard outcome measures (eg, mortality ratios), although Ramirez et al^3^ found that PSRSs led to a decrease in observed near misses and adverse events in their institution. This finding emphasizes the need for further examination of voluntary PSRSs.

In the current literature, 2 major challenges are apparent. First, many systems are experiencing volume overload, because a single health care center may receive thousands of reports per year.^2^ Owing to lack of time and available personnel, a thorough investigation into all these events is impossible. Second, the cost associated with evaluating patient safety reports is not insignificant. One study from the United Kingdom^4^ found the cost per incident report form completed was £337.16 (US $466.70). However, even when those challenges are lessened, the voluntary nature of event reporting could be subject to the implicit bias of the reporter. Reported events, especially those citing disruptive behavior, may have elements of bias in how incidents are described in the report and what events are reported. This can lead to unforeseen negative consequences such as mistrust, deterioration of team communication, and hinderance of career advancement.

To date, the literature surrounding PSRSs has focused on efficiency, efficacy, and patterns in the types of events reported.^2,3,4^ However, a paucity of literature exists examining content of patient safety reports as it relates to identities that influence power dynamics—namely, gender, race and ethnicity, and job position. By examining the content of PSRS reports, we hope to ascertain whether there is bias in how events are reported based on the race and ethnicity, gender, or faculty rank of the person who is the subject of the event report.

## Methods

The PSRS available in the Stanford Health Care System is the Stanford Alert for Events (SAFE). It allows for any hospital employee to report events that might impede patient safety and quality of care. Reporters using the SAFE system must identify the subject of their complaint when recounting disruptive behavior. Given this requirement, inclusion requirements for this study were (1) a report involving perceived disruptive behavior with a faculty physician as the subject and (2) a SAFE report that specifically addressed the behavior and/or actions of the faculty physician. Of note, reports that focused solely on patient-facing issues (without mention of physician actions and/or behavior) were excluded from this study. Focusing on physician faculty increased the likelihood of determining race and ethnicity and gender of the subject of the report, because Stanford University School of Medicine, Stanford, California, has documented self-reported demographic data on faculty. In addition, using physician faculty allowed for conducting an analysis by faculty rank. We did not examine the demographic information or job position of the author of the SAFE report because events are often filed anonymously. This study was deemed exempt from evaluation and informed consent by the Stanford University School of Medicine Institutional Review Board. This study followed the Consolidated Criteria for Reporting Qualitative Research (COREQ) reporting guideline.

Events reported from March 2011 to February 2020 were collected from the adult hospital. Stanford received 800 to 900 reports per month during this time. During the study period, 501 reports total met the inclusion criteria listed above. We excluded 100 reports owing to incompleteness of report information and/or missing physician demographic information. A qualitative analysis was performed on the remaining 401 reports representing 187 physicians. A unique numeric identifier was assigned to each of the 401 event reports. The qualitative analysis was then completed without identification of gender, race and ethnicity, and faculty rank data of the physician. Separately, gender, race and ethnicity, and faculty rank data were merged with physician names using the self-reported Stanford University School of Medicine demographic data on gender, race and ethnicity, and faculty rank data. Once the demographic data were merged, physician identifier information was removed from the report. The qualitative analysis of the event reports and the physician demographic data were then merged based on the previously assigned unique event numeric identifier.

Two coders manually developed a coding scheme consisting of 7 themes through an inductive thematic approach in consultation with 2 of the investigators (É.B. and M.F.). In line with COREQ guidelines, the coders were qualitative researchers outside the field of medicine who were blinded to the demographic characteristics of the reported physicians. The 401 reports coded represented all those who reported perceived disruptive behavior on the part of faculty physicians during the study period at the Stanford University School of Medicine using the SAFE reporting system. The initial 50 of the 401 reports were used to identify themes relevant to the analytic objective of categorizing the different types of incidents for which physicians were reported. The coders searched for repetition of incident types across reports to identify themes.^5^ Emerging themes discovered during this early analysis were documented independently and used by the coders to create an initial coding scheme. The coders independently applied this scheme to the subsequent 50 reports to assess the validity of the codes when applied to new reports. Following this analysis, the research team refined the definitions of each theme and solidified theme boundaries by documenting distinctions between related themes using a constant comparison method.^5^ With the coding scheme finalized, the first 100 reports that were analyzed during the theme discovery stage were independently recoded by the 2 coders with no more than 2 themes: a primary and a secondary theme. The research team reconvened to resolve discrepancies and ensure that 100% agreement for all codes was achieved. Quality assurance meetings occurred after every 50 reports to resolve any discrepancies and achieve 100% agreement for all codes. An additional coder was trained to expedite report coding.

Seven themes emerged from the data: inappropriate communication, verbal abuse, physical intimidation, process issues, ignoring or omitting procedures, conversational conduct, and lack of communication (Table 1). Each report was also assigned a single severity level of 1 (lowest) for distressing events, 2 for harsh events, or 3 (highest) for egregious events (Table 1). The examples listed in Table 1 were chosen because they are particularly ripe for differential reporting based on identities. Furthermore, these examples underscore reports that may produce low-yield safety benefit and should not be viewed as examples of appropriate or high-yield safety reports of well-functioning PSRSs.

**Table 1.** Theme and Severity Coding Scheme

Theme or code	Description of incidents	Example
Theme		
Inappropriate communication	Physicians were perceived as rude, made snide comments, or expressed inappropriate body language	Dr X told the patient, “You know I’m giving up my night before Thanksgiving to be here” and immediately left the room. The patient stated, “I’m afraid to make [them] angry again because I don’t want [them] to take away my pain medicine.”
Verbal abuse	Physicians yelled, used profanity, or threatened a colleague’s employment	Dr X yelling at OR staff because [their] surgery wasn’t going well, as well as demeaning comments to the anesthesiology resident “how could you not get the spinal in 45 minutes.” Overall unprofessional behavior.
Physical intimidation	Physician displayed inappropriate physical behavior such as looming over someone or throwing equipment	Throughout the day, if I was not holding a retractor in place where [they] placed it, Dr X would roughly slap and grab my hand to pull on the retractor to reposition it. [They] would also raise [their] voice to tell me I was holding the retractors wrong.
Process issue	Structural errors were present. Examples include inadequate staff training or pager numbers listed incorrectly in the system	Duplicate request for post-op pelvis [study]. Also ordered to wrong resource, North x-ray got one and so did PACU. Patient almost got x-rayed twice.
Ignoring or omitting procedures	Physicians did not follow standard procedure such as timeouts or adhering to proper sterile technique	Dr X scrubbed and performed this patient’s surgery while wearing [their] wedding band. [They were] asked many times not to do this, as it is in violation of Stanford’s jewelry policy for scrubbed personnel. [They] refused to comply. This is an infection risk for the patient.
Conversational conduct	Physicians displayed verbal conduct that is not suited for the workplace but did not involve communication issues directed at others. Examples include physicians that discussed patient cases loudly or had an extended personal phone call	Drs X, Y, and Z were in A5 discussing a case for the following day. They were asked to leave the room by the RN and then by the ST because they were delaying patient care. I was called to the room and asked the MDs to leave twice, I told them there was an empty room available to measure beds if needed.
Lack of communication	Inadequate or incomplete communication from the physician	Dr X left the operating room stating, ”I am taking a break.” [They] returned at 1643. In [the MD’s] absence the surgery did not proceed. Per manager review: Surgeon left surgery for the total of 56 min. Patient under anesthesia with no activity.
Severity level		
1 (Distressing events)	Sarcastic comments, not answering a page, or eye rolling	MD kept stating, “I don’t care what excuse you have, stop arguing with me,” in a very loud manner and disrespectful tone. RN stated that [they] understood [the MD’s] concern but would not talk to the MD in [their] current tone of voice…. MD hung up on RN.
2 (Harsh events)	Yelling, public humiliation, or threatening discourse	Dr X yelling at OR staff because [their] surgery wasn’t going well. As well as demeaning comments to the anesthesiology resident “how could you not get the spinal in 45 minutes.” Overall unprofessional behavior.
3 (Egregious events)	Physical altercations, forcing a procedure onto a patient, or repeated unprofessional behavior	Dr X was yelling, using inappropriate language and very confrontational. [They] were standing over me and kept pointing [their] finger in my face despite my multiple requests to stop. In all my 18 y at Stanford I have never been treated so unprofessionally by anyone, let alone a physician.

Abbreviations: MD, medical doctor; OR, operating room; PACU, postanesthesia care unit; RN, registered nurse; ST, surgical technologist.

After all reports were coded for severity and theme, we conducted analyses by demographic categories of gender, race and ethnicity, and faculty rank. The total percentage of the 401 reports, categorized by theme and severity, was calculated for each demographic category (gender, race and ethnicity, and faculty rank) (Table 2). The calculated total percentages by demographic category served as our reference point and/or benchmark for the subsequent analysis (Tables 3, 4, and 5). Statistical summaries of the differences were calculated in 2 ways: (1) a series of 1-sample χ^2^ statistical tests of the null that the complaint distribution across race was identical to the distribution of physician race summarized in Table 4 and (2) an indicator of a large departure was set before looking at the data (ie, a priori). As there is no standardized effect size in categorical variables (ie, there is no equivalent of a Cohen *d*, owing to the nondirectionality of a χ^2^ test), we used the following criterion for a meaningfully large effect size: if the percentage of reports assigned a certain theme or severity level for a given demographic was 3% or more above the benchmark, the specific theme or severity level was deemed to have disproportionate representation for the given demographic. The 3% threshold, chosen a priori, was arbitrary (similar to the convention of the *P* value of .05) to identify a meaningful departure from the expected value, but the values themselves are reported in Tables 3, 4, and 5.

**Table 2.** Physician Demographics^a^

Characteristic	No. (%) of physicians	
	Stanford University School of Medicine faculty (n = 1480)^b^	Physicians reported via SAFE (n = 187)^c^
Race and ethnicity		
African American	25 (1.7)	4 (2.1)
Asian	390 (26.3)	49 (26.2)
Hispanic or Latinx	59 (4.0)	7 (3.7)
White	834 (56.3)	108 (57.7)
Declined to state	151 (10.2)	19 (10.2)
Other^d^	21 (1.4)	0
Gender		
Female	636 (43.0)	49 (26.2)
Male	844 (57.0)	138 (73.8)

Abbreviation: SAFE, Stanford Alert for Events.

^a^ Includes nonpediatric clinical faculty with full-time equivalents of greater than 0.49.

^b^ We used the midpoint year of 2015 to account for the entire study period.

^c^ From March 2011 to February 2020.

^d^ Includes faculty with identities that did not fall into 1 of the 4 largest categories (eg, multiracial).

**Table 3.** Themes and Severity by Gender

Report characteristic (No. of reports)	Physician gender, No. (%) of reports^a^		*P* value
	Female	Male	
Gender (401)	88 (21.9)	313 (78.1)	<.001
Theme (516)			
Inappropriate communication (177)	46 (26.0)^b^	131 (74.0)	<.001
Ignoring or omitting procedures (103)	19 (18.4)	84 (81.5)^b^	<.001
Verbal abuse (95)	22 (23.1)	73 (76.8)	<.001
Lack of communication (50)	13 (26.0)^b^	37 (74.0)	.02
Process issue (38)	6 (15.8)	32 (84.2)^b^	.001
Physical intimidation (38)	5 (13.1)	33 (86.8)^b^	<.001
Conversational conduct (15)	6 (40.0)^b^	9 (60.0)	>.99
Severity (401)			
1 (Distressing) (119)	30 (25.2)^b^	89 (74.8)	<.001
2 (Harsh) (245)	50 (20.4)	195 (79.6)	<.001
3 (Egregious) (37)	8 (21.6)	29 (78.4)	.01

^a^ Percentages are based on 401 coded reports with 516 coded themes. All percentages are row percentages.

^b^ Indicates difference of at least 3% compared with the benchmark demographic characteristic listed (636 [43.0%] female and 844 [57.0%] male physicians).

**Table 4.** Themes and Severity by Race and Ethnicity

Report characteristic (No. of reports)	Physician race and ethnicity, No. (%) of reports^a^				*P* value^b^
	African American	Asian	Latinx	White	
Race and ethnicity (401)	19 (4.7)	83 (20.7)	22 (5.5)	236 (58.9)	<.001
Theme (516)					
Inappropriate communication (177)	5 (2.8)	37 (20.9)	9 (5.1)	101 (57.1)	.33
Ignoring or omitting procedures (103)	5 (4.9)	18 (17.5)	5 (4.9)	67 (65.0)^c^	.03
Verbal abuse (95)	2 (2.1)	22 (23.1)	3 (3.1)	59 (62.1)^c^	.79
Lack of communication (50)	9 (18.0)^c^	12 (24.0)^c^	3 (6.0)	25 (50.0)	<.001
Process issue (38)	4 (10.5)	9 (23.7)^c^	2 (5.3)	19 (50.0)	.001
Physical intimidation (38)	1 (2.6)	2 (5.3)	3 (7.9)	28 (73.7)^c^	.11
Conversational conduct (15)	0	4 (26.7)^c^	2 (13.3)^c^	9 (60.0)	.41
Severity (401)					
1 (Distressing) (119)	9 (7.6)	32 (26.9)^c^	5 (4.2)	61 (51.3)	<.001
2 (Harsh) (245)	8 (3.3)	47 (19.2)	14 (5.7)	151 (61.6)	.03
3 (Egregious) (37)	2 (5.4)	4 (10.8)	3 (8.1)	24 (64.9)^c^	.04

^a^ Percentages are based on 401 coded reports with 516 coded themes. All percentages are row percentages and include in the denominator 41 reports and 56 coded themes about 19 physicians for whom race and ethnicity were declined to state or unknown. Data from these reports are not shown.

^b^ Generated from χ^2^ tests by comparing the race and ethnicity distribution in each theme with the marginal race distribution in the physician workforce.

^c^ Indicates difference of at least 3% compared with the benchmark demographic characteristic listed (25 [1.7%] for African American, 390 [26.3%] for Asian, 59 [4.0%] for Latinx, and 834 [56.3%] for White physicians).

**Table 5.** Themes and Severity by Faculty Rank

Report characteristic (No. of reports)	Faculty rank, No. (%) of reports^a^				*P* value
	Clinical instructor (140 [9.5%])	Assistant professor (577 [39.0%])	Associate professor (340 [23.0%])	Professor (423 [28.5%])	
Faculty rank (n = 401)	19 (4.7)	102 (25.4)	124 (30.9)	156 (38.9)	<.001
Theme (n = 516)					
Inappropriate communication (177)	10 (5.6)	49 (27.7)	60 (33.9)^b^	58 (32.8)	<.001
Ignoring or omitting procedures (103)	6 (5.8)	25 (24.3)	29 (28.1)	43 (41.7)	.003
Verbal abuse (95)	3 (3.1)	29 (30.5)^b^	18 (18.9)	45 (47.4)^b^	<.001
Lack of communication (50)	5 (10.0)^b^	10 (20.0)	24 (48.0)^b^	11 (22.0)	<.001
Process issue (38)	4 (10.5)^b^	6 (15.8)	16 (42.1)^b^	12 (31.6)	.01
Physical intimidation (38)	0	9 (23.7)	11 (28.9)	18 (47.4)^b^	.41
Conversational conduct (15)	0	3 (20.0)	4 (26.7)	8 (53.3)^b^	.11
Severity (401)					
1 (Distressing) (119)	8 (6.7)	29 (24.4)	52 (43.7)^b^	30 (25.2)	<.001
2 (Harsh) (245)	9 (3.7)	63 (25.7)	62 (25.3)	111 (45.3)^b^	<.001
3 (Egregious) (37)	2 (5.4)	10 (27.0)	10 (27.0)	15 (40.5)	.26

^a^ Percentages are based on 401 coded reports with 516 coded themes. All percentages are row percentages.

^b^ Indicates difference of at least 3% compared with the benchmark demographic characteristic listed (140 [9.5%] for clinical instructor, 577 [39.0%] for assistant professor, 340 [23.0%] for associate professor, and 423 [28.6%] for professor).

## Results

Included reports represented 187 physicians (138 [73.8%] male and 49 [26.2%] female). In terms of race and ethnicity, 4 physicians (2.1%) were African American, 49 (26.2%) were Asian, 7 (3.7%) were Hispanic or Latinx, 108 (57.7%) were White, and 19 (10.2%) declined to state. All 401 reports were assigned a primary theme (theme 1), and 115 of 401 reports were assigned a secondary theme (theme 2), for a total of 516 combined themes. A single severity level was assigned to each report, totaling 401 severity levels. Of the 187 physicians in the data set, 111 (59.3%) received 1 report, 65 (34.7%) received 2 to 6 reports, 10 (5.3%) received 7 to 11 reports, and 1 (0.5%) received 15 reports.

The overall demographics for the Stanford University School of Medicine faculty for 2015 (chosen as a midpoint for the study period) are listed in Table 2. Tables 3, 4, and 5 list the total percentages of the 401 reports for each demographic category (gender, race and ethnicity, and faculty rank), including benchmark, by theme and severity.

### Gender

Female physicians had disproportionate representation among reports for inappropriate communication, lack of communication, conversational conduct, and reports categorized as severity level 1. Male physicians had disproportionate representation among reports for ignoring/omitting procedures, process issues, and physical intimidation (Table 3).

### Race and Ethnicity

Asian physicians had disproportionate representation in reports coded as lack of communication, process issues, conversational conduct, and severity level 1. African American physicians similarly had disproportionate representation for lack of communication and process issue events. Latinx physicians had disproportionate representation for conversational conduct. White physicians had disproportionate representation for ignoring or omitting procedures, verbal abuse, physical intimidation, and severity level 3 reports (Table 4).

### Faculty Rank

Clinical instructors had disproportionate representation for lack of communication and process issues events. Assistant professors had disproportionate representation for verbal abuse. Associate professors had disproportionate representation for inappropriate communication, lack of communication, process issues, and severity level 1 reports. Professors had disproportionate representation for verbal abuse, physical intimidation, conversational conduct, and severity level 2 reports (Table 5).

## Discussion

Following the recommendations from the Institutes of Medicine, various legislations and accreditation organizations mandated health care organizations have a means to report errors that may compromise patient safety and quality of care.^2^ Patient safety reporting systems help health care organizations fulfill these requirements and are designed to proactively “improve quality of care and patient safety.”^6^^(p1)^ Types of events reported consist of any incident that could have resulted or resulted in patient harm or death, including unprofessional or disruptive staff behavior.^6^ Disruptive behaviors impede communication and collaboration, both of which are vital for quality patient care in the current era of multidisciplinary teams.^6^ Although it is important to report any events or behaviors that are not conducive to a safe work environment, it has been shown that PSRSs are subject to reporting bias owing to their reliance on voluntary reporting.^2^ However, the question of whether PSRSs are subject to implicit biases remains unanswered.

There are multiple accounts in the literature pertaining to discrimination experienced by physicians based on gender, race and ethnicity, and other personal characteristics.^7^ Moreover, when individuals do not adhere to their expected roles, they may be subject to negative stereotyping.^8^ This concept was explored by Fassiotto et al^8^ when they examined the voluntary medical trainee evaluations of physician faculty. Their findings revealed that in certain specialties, female physicians received lower median scores compared with male physicians.^8^ Our study aimed to determine whether similar biases existed in the reporting of SAFE events based on the gender, race and ethnicity, and academic faculty rank of reported physicians at a single academic medical center.

All employees sign and are expected to follow a professional code of conduct, thus creating a set standard of behavior among employees. The findings of the present study therefore suggest that female physicians and physicians who are members of racial and ethnic minority groups may be subject to different standards and negative stereotyping in reported instances of communication misconduct. In addition, female and Asian physicians were more likely to be reported for events of the lowest severity, whereas White physicians were more likely reported for events of the highest severity. Although these findings did not meet our threshold (≥3% of the benchmark), African American physicians were more likely to be reported for events of the lowest severity (2.9% above benchmark), and Latinx physicians were more likely to be reported for events of the highest severity (2.6% above benchmark). These results suggest that there may be a lower threshold for filing an event report when the physician subject of the report is female, African American, or Asian. The implication of this difference is important because it not only suggests a level of implicit bias in communications with female, African American, and Asian faculty but also has repercussions for career advancement. In our analysis of faculty rank, clinical instructors had trends similar to female physicians and members of racial and ethnic minority groups, whereas those at the professor level had trends similar to those of White faculty. This finding highlighted the prevalence of female physicians and those who were members of racial and ethnic minority groups at lower academic ranks. A previous study found disparities in the promotion of African American academic surgeons at the assistant professor level as well as lower retention rates for academic surgeons who are members of underrepresented minority groups.^9^ SAFE reports are often considered when making recommendations in the promotion process, and if biases in reporting systems exist, they could play a role in female faculty and those who are members of racial and ethnic minority groups being disproportionately promoted at lower rates.

Settles et al^10^ examined this notion of epistemic exclusion in a study of underrepresented minority groups in academia, addressing challenges where institutional systems intersect with individual bias and subsequently devalue the scholarship of members of racial and ethnic minority groups and women. Although this study specifically addressed devaluing scholarship and research efforts in academia as a means to stall career advancement, this term could apply in the realm of health care. A possible example is given by Smith,^11^ who cites an incident in which she was hired to coach an emergency department physician after the physician received complaints from a nurse stating the physician had anger management issues.^11^ After her investigation, Smith concluded the physician “did not have anger management problems. She had frustration management problems.”^11^ In this example, the physician was labeled as having an anger management problem, creating a narrative of a disruptive physician. Over time, SAFE reports with similar narratives could be used to devalue the clinical skill of female and underrepresented minority group physicians, possibly impeding promotion.

Patient care is currently steeped in multidisciplinary teams that require effective communication. A recent study from our pediatric hospital found “the most concerning care gaps and failures in medical decision-making were due to challenges with communication…when multiple subspecialty groups”^12^^(p2)^ were involved. Implicit bias has been shown to cause further breakdown of communication and interdisciplinary collaboration due to perceived power imbalances.^13^ Moreover, implicit bias was cited as contributing to poor team performance in critical clinical situations, directly causing patient harm.^13^ Physicians subjected to implicit bias have also been shown to be at higher risk for burnout.^13^ Thus, PSRSs may negatively affect the well-being of physicians and unintentionally reinforce poor team communication if influenced by implicit bias.

In the US, underrepresented minority groups endure inequities due to systemic discrimination. As discrimination is not always overt, it can take the form of implicit bias, which can lead to unfavorable assessments.^14^ These unfavorable assessments adversely influence institutional processes, thereby reinforcing systemic discrimination and contributing to epistemic exclusion. Patient safety reporting systems, although well intentioned, may indirectly contribute to systemic discrimination because PSRSs are subject to reporter bias and their implicit bias. As such, health care systems should exercise caution when using event reporting in career advancement decision making. Consequently, we propose creating a separate system to review event reports involving issues with communication or nonsevere inappropriate behavior that allows for the following:

- reviewers to devote the appropriate amount of time to thoroughly investigate these events;
- the involved parties to be brought together to openly and constructively discuss the event;
- methods of conflict resolution that enhance communication between involved parties to be used and taught; and
- proactive approaches that focus on effective team building to prevent or decrease the occurrence of future events.

Sandborg et al^12^ describe a similar model that allowed for the peer-review process to occur in 1 of 3 committees depending on the nature of the report. Using 3 committees instead of the one-size-fits-all approach of a single committee enabled use of a process that simultaneously tackled health care professionals’ competence and facilitated learning.^13^ Amending the current system in these ways could improve colleague interactions (aiding to dispel negative stereotypes) and decrease the number of events reported, resulting in cost savings of report analysis.

### Limitations

There are several limitations to this study. First, we only included data from an adult hospital at a single academic institution, which affects the generalizability and reproducibility of our findings. Second, our study population included a small sample size of physicians who are members of racial and ethnic minority groups. We also were unable to categorize the race and ethnicity of the physicians representing 41 reports (10.2%), which further affected our analysis for the demographic of race and ethnicity and precluded intersectional analyses (eg, women by race and ethnicity). Third, our coding scheme allowed for multiple theme categorization with the most severe element guiding the severity scoring; therefore, all elements of the complaint may not have been captured. Finally, there is a statistical limitation to analyzing the existing data, because the reporting mechanism censors a subset of reports. Here we use the technical statistical term *censoring* to mean that not all events that could generate SAFE were observed in our data set, therefore the data set did not allow for a full statistical analysis of variation in SAFE rates across groups. One hypothesis consistent with the observed reporting patterns is that physicians with identities that benefit from a perceived power differential are more likely to have encounters not reported that might otherwise have been reported through the SAFE system. A reporting system that uses random sampling rather than voluntary reports would be more likely to generate a data set that accurately reflects encounters in this setting.

## Conclusions

The findings of this qualitative study of a single academic institution’s PSRS suggest that there may be a differential threshold for reporting low-severity events when the physician subject of the report is female or a member of a racial or ethnic minority group vs male or White, respectively. Considering the potential serious repercussions of bias in PSRSs, future studies should examine PSRSs at other institutions, evaluate different processes that focus on improved communication and/or team building, and determine whether there is an association between being the subject of a patient safety report and the rate of promotion among female faculty and those who are members of racial and ethnic minority groups.

## References

[1] Leape LL. Patient safety in the era of healthcare reform. Clin Orthop Relat Res. 2015;473(5):1568-1573. doi:10.1007/s11999-014-3598-6 24748068 PMC4385369

[2] Pronovost P, Morclock L, Sexton B, . Improving the value of patient safety reporting systems. August 2018. Accessed December 31, 2020. https://www.ahrq.gov/downloads/pub/advances2/vol1/Advances-Pronovost_95.pdf

[3] Ramírez E, Martín A, Villán Y, ; SINOIRES Working Group. Effectiveness and limitations of an incident-reporting system analyzed by local clinical safety leaders in a tertiary hospital: prospective evaluation through real-time observations of patient safety incidents. Medicine (Baltimore). 2018;97(38):e12509. doi:10.1097/MD.0000000000012509 30235764 PMC6160204

[4] Sinclair A, Guérin A, Robin C, Dey P. Investigating the cost and efficiency of incident reporting in a specialist paediatric NHS hospital and impact on patient safety. Eur J Hosp Pharm. 2017;24(2):91-95. doi:10.1136/ejhpharm-2016-000926 31156911 PMC6451566

[5] Guest G, MacQueen K, Namey E. Themes and codes. In: Guest G, MacQueen K, Namey E, eds. Applied Thematic Analysis. SAGE Publications; 2012:49-78.

[6] Patient Safety Systems. CAMH January 2019 E-dition. July 1, 2019. Accessed April 19, 2022. https://www.jointcommission.org/-/media/tjc/documents/standards/ps-chapters/20190701_2_camh_04a_ps.pdf

[7] Frost M. How to handle biased patients. ACP Hospitalist. June 15, 2020. Accessed November 16, 2020. https://acphospitalist.acponline.org/search/index.fcgi?query=how+to+handle+biased+patients

[8] Fassiotto M, Li J, Maldonado Y, Kothary N. Female surgeons as counter stereotype: the impact of gender perceptions on trainee evaluations of physician faculty. J Surg Educ. 2018;75(5):1140-1148. doi:10.1016/j.jsurg.2018.01.011 29402668

[9] Abelson JS, Wong NZ, Symer M, Eckenrode G, Watkins A, Yeo HL. Racial and ethnic disparities in promotion and retention of academic surgeons. Am J Surg. 2018;216(4):678-682. doi:10.1016/j.amjsurg.2018.07.020 30086831

[10] Settles I, Jones M, Buchanan N, Dotson K. Epistemic exclusion: scholar(ly) devaluation that marginalizes faculty of color. J Divers High Educ. Published online March 2, 2020. doi:10.1037/dhe0000174

[11] Smith S. Disruptive behavior in healthcare. Becker’s Hospital Review. January 3, 2017. Accessed December 14, 2020. https://www.beckershospitalreview.com/hospital-management-administration/disruptive-behavior-in-healthcare.html

[12] Sandborg CI, Hartman GE, Su F, . Optimizing professional practice evaluation to enable a nonpunitive learning health system approach to peer review. Pediatr Qual Saf. 2020;6(1):e375. doi:10.1097/pq9.0000000000000375 33409427 PMC7781295

[13] Sanchez E, Tran-Reina M, Ackerman-Barger K, Phung K, Molla M, Ton H. Implicit biases, interprofessional communication, and power dynamics. April 29, 2020. Accessed June 6, 2021. https://psnet.ahrq.gov/web-mm/implicit-biases-interprofessional-communication-and-power-dynamics

[14] Kirwan Institute for the Study of Race and Ethnicity. Understanding implicit bias. May 29, 2012. Accessed December 7, 2020. https://kirwaninstitute.osu.edu/article/understanding-implicit-bias

